# High *Toxocara cati* prevalence in wild, free-ranging Eurasian lynx (*Lynx lynx*) in Finland, 1999–2015

**DOI:** 10.1016/j.ijppaw.2022.02.004

**Published:** 2022-02-09

**Authors:** Miisa Virta, Otso Huitu, Juha Heikkinen, Katja Holmala, Pikka Jokelainen

**Affiliations:** a Natural Resources Institute Finland, Latokartanonkaari 9, 00790, Helsinki, Finland; b Faculty of Biological and Environmental Sciences, University of Helsinki, Viikinkaari 1, 00014, Helsinki, Finland; c Natural Resources Institute Finland, Korkeakoulunkatu 7, 33720, Tampere, Finland; d Infectious Disease Preparedness, Statens Serum Institut, Artillerivej 5, DK-2300, Copenhagen, Denmark; e Faculty of Veterinary Medicine, University of Helsinki, Agnes Sjöberginkatu 2, 00014, Helsinki, Finland

**Keywords:** Eurasian lynx, Population growth, Host-parasite dynamics, *Toxocara cati*

## Abstract

In Finland, free-ranging Eurasian lynx (*Lynx lynx*) population has grown from 30 to 40 individuals to 2800 individuals since the species became partly protected in 1962. Changes in host population size are known to have an impact on host-parasite dynamics, and the Eurasian lynx population in Finland provides a unique opportunity for studying the potential effects of dramatic population increase and expansion of a solitary apex predator on their parasite prevalence and abundance. *Toxocara cati* is a zoonotic gastrointestinal parasite infecting domestic cats and wild felids worldwide. We studied *T. cati* infection prevalence and worm burden in 2756 Eurasian lynx individuals from Finland, covering the years 1999–2015. *Toxocara cati* worms that had been collected from intestinal contents were identified based on morphology. We performed regression analyses to investigate possible associations of age, sex, and host population density with *T. cati* infection. We found *T. cati* from 2324 (84.3%, 95% confidence interval 82.9–86.0) of the examined lynx. Each year, the infection prevalence was higher than 75% and not density dependent. The parasites were strongly aggregated, with older individuals harboring fewer *T. cati* than younger ones did. Old females aged 9–15 years had higher *T. cati* abundance than males of the same age group. Our results indicate that *T. cati* was a common and abundant parasite of Eurasian lynx throughout the study period, regardless of the changing population size and density.

## Highlights

• 84% of 2756 Eurasian lynx (*Lynx lynx*) from Finland had *Toxocara cati* infection.
• The prevalence was high throughout 1999–2015, while lynx population size tripled.
• Old female lynx had significantly higher *Toxocara cati* abundance than old males.

## Graphical abstract

**Figure:**
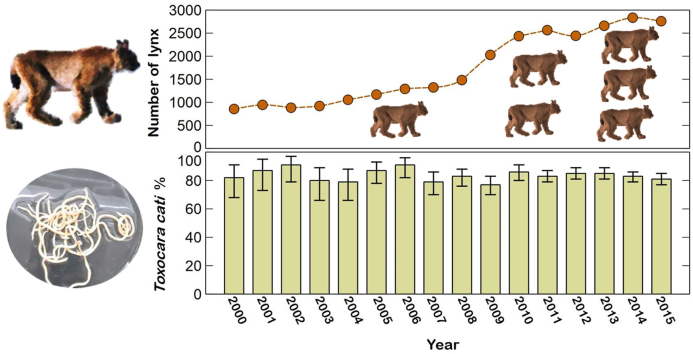

## 1 Introduction

The zoonotic gastrointestinal nematode *Toxocara cati* is a common parasite infecting domestic cats as well as wild felids worldwide (Sprent, 1956; Bagrade et al., 2003; Valdmann et al., 2004; Sommerfelt et al., 2006; Zibaei et al., 2007; Okulewicz et al., 2012; Gallas and Silveira, 2013; Otranto and Deplazes, 2019; Hou et al., 2020; Figueiredo et al., 2021; Ursache et al., 2021). In Finland, the prevalence of *T. cati* in domestic cats was estimated to be 5.4%, based on detection of eggs of the parasite in feces (Näreaho et al., 2012). *Toxocara cati* is a common parasite also of free-ranging Eurasian lynx (*Lynx lynx*) in the country (Deksne et al., 2013). Data on *T. cati* prevalence in both domestic cats and wild felids are needed to understand the circulation of the parasite and the zoonotic and cross-species infection risks.

Both felids and humans can become infected with *T. cati* by ingesting embryonated eggs of the parasite or by eating tissues of paratenic hosts (Sprent, 1956; Overgaauw et al., 1997; Fisher, 2003; Coati et al., 2004; Davidson et al., 2012; Strube et al., 2013). Moreover, kittens of domestic cats can become infected via milk (Swerczek et al., 1971).

Parasite prevalence and abundance tend to follow host population size and density (May and Anderson, 1979; Dobson and Hudson, 1992; Arneberg et al., 1998). It is assumed that host-parasite dynamics follow the Lotka-Volterra predator-prey model: changes in the population size of a prey or a host are followed by changes in the population size of a predator or a parasite (Anderson and May 1978; Dobson and Hudson, 1992). However, there are only few studies on how population increase and expansion of large carnivores has affected their parasites. For example, in Germany, parasite prevalence and diversity in wolves increased with advancing recolonization process (Leshniak et al., 2017).

Age and sex of the host are also known to have an impact on parasite abundance and intensity (Kołodziej-Sobocińska, 2019). Juveniles still developing acquired immunity can be more heavily infected and have higher prevalence than adults (Hudson and Dobson, 1995; Gates and Nolan, 2009; Reinemeyer and Nielsen, 2017). In older individuals, declining immune function may explain higher parasite burden (Froy et al., 2019). Sex-biased parasitism is common in many mammal species (Moore and Wilson, 2002; Krasnov et al., 2005). The differences in immunity, prey selection, feeding habits, home range size, and hormones can to some extent explain differences in parasitism between sexes (Poulin, 1996; Hillegass et al., 2008; Friesen et al., 2015; Kojola et al., 2017; Albery et al., 2020). In females, estrogen stimulates immunity, whereas in males the major androgen testosterone is an immunosuppressive hormone and therefore supports male-biased parasitism (Schuurs and Verheul, 1990; Poulin, 1996).

Parasites often exhibit an aggregated distribution within their host population – a few hosts harbor many parasites, whereas most hosts have none or only a few parasites (Crofton, 1971; Shaw and Dobson, 1995; Morand and Krasnov, 2008). Factors such as age and season can influence the degree of parasite aggregation (Hudson et al., 1992; Boag et al., 2001; Saeed and Kapel, 2006; Sherrard-Smith et al., 2015). The effect of age may be related to immunity, and the seasonal changes to weather conditions, which can affect infective stages of the parasites or intermediate hosts (Hudson et al., 1992; Boag et al., 2001; Fromont et al., 2001; Newey et al., 2005).

Previous studies have shown that Eurasian lynx in Finland are hosts to protozoa including *Toxoplasma gondii* and *Isospora* sp., nematodes including *T. cati* and *Trichinella* spp., and cestodes including *Taenia* spp. and *Mesocestoides* sp. (Oksanen et al., 1998; Airas et al., 2010; Deksne et al., 2013; Jokelainen et al., 2013; Lavikainen et al., 2013; Haukisalmi et al., 2016; Kojola et al., 2017). In particular, *T. cati* was found to be a highly prevalent parasite, found from intestines of 92.9% of investigated Eurasian lynx (Deksne et al., 2013), which is in line with findings from other countries (Bagrade et al., 2003; Valdmann et al., 2004; Kołodziej-Sobocińska et al., 2018).

The Eurasian lynx population size in Finland decreased in the early 20th century due to hunting (Pulliainen and Rautiainen, 1999). The species became partly protected in 1962, at a time when the estimated population size was only 30–40 individuals, predominately living in south-eastern Finland (Pulliainen and Rautiainen, 1999). The protection together with a change in prey species to increasingly available white-tailed deer (*Odocoileus virginianus*) and European roe deer (*Capreolus capreolus*) enabled the Eurasian lynx population size to increase, reaching approximately 855 individuals in 1995 (Pulliainen, 1981) and 2800 individuals in 2013 (Natural Resources Institute Finland, 2014). In eastern Finland, hares (Leporidae) and grouse (Tetraonidae) are the main prey of Eurasian lynx, whereas in southern and south-western Finland the typical diet includes equal proportions of hares and small cervids (Cervidae) (Pulliainen et al., 1995).

The history of Eurasian lynx population in Finland provides an opportunity to study the potential effects of dramatic population increase and expansion of a solitary apex predator on their parasite prevalence and abundance. The aim of this study was to describe *T. cati* prevalence and abundance in Eurasian lynx in Finland over a 16-year study period, while the lynx population size increased. We hypothesized that the increase in host population size and density would be positively associated with *T. cati* prevalence and abundance. We estimated the prevalence by year and investigated whether age and sex of the lynx were associated with having the infection. We hypothesized that young Eurasian lynx would have higher *T. cati* prevalence than older ones, and that males would have higher prevalence than females.

## 2 Materials and methods

### 2.1 Study setting and design

The study was a retrospective observational study. The data included parasite worms collected from intestines of 2756 Eurasian lynx that had been legally hunted or had died in e.g. car collisions in Finland in 1999–2015. No animals were killed for the purpose of this study. All procedures were performed in compliance with relevant laws and guidelines.

The Eurasian lynx is protected in Finland. Hunting is permitted for damage control and for population management purposes. Yearly quota is set by Ministry of Forestry and Agriculture, and permits are annually issued by Finnish Game Agency for the 15 game managements districts (GMD), based on the estimated lynx population size in each GMD. Population size estimation is based on observed number of litters (Natural Resources Institute Finland, 2016). The hunting period is from the beginning of October to the end of February in reindeer herding area, and from the beginning of December to the end of February in other areas. Data from 14 GMDs were included in the present study; GMD covering Lapland in northern Finland was excluded due to limited sample size and non-comparable estimates of population size.

Frozen carcasses of Eurasian lynx were sent by hunters and officials to Taivalkoski research station of Natural Resources Institute Finland. The carcasses were dissected and the whole gastrointestinal tract was searched for parasitic worms. The intestinal contents were rinsed with water on a fine mesh sieve, and all visible gastrointestinal parasitic worms were collected. Nematodes were separated from cestodes based on morphology, and counted. The worms were stored in 70% ethanol at +8 °C temperature. The age of each individual lynx was determined based on analysis of cementum annuli of their teeth by the Matson Laboratory, Montana, USA (Matson, 1981).

We identified *T. cati* parasites based on the key morphological features with focus on their anterior end, in particular the shape and position of cephalic alae (Sprent, 1956), and recorded their absence or presence and numbers. Cestode absence/presence was included in the analyses as a dichotomous variable to investigate co-infections.

### 2.2 Statistical analyses

The presence of *T. cati* was treated as a binary variable (absence/presence). We report prevalence, abundance and intensity. Apparent *T. cati* infection prevalence was defined by dividing the number of Eurasian lynx that had *T. cati* infection by the number of examined Eurasian lynx. Abundance denotes the number of *T. cati* parasites in any given lynx individual, while intensity denotes the number of *T. cati* parasites in an infected lynx (Bush et al., 1997).

Logistic regression was used to analyze the relationship between *T. cati* presence and the age of the lynx (continuous variable), the sex of the lynx, the year when the lynx was hunted, the presence of cestodes, and GMD-level lynx population density index. The lynx population density index was calculated by dividing the annual Eurasian lynx population size estimate of each GMD by the area, in km^2^, of the GMD.

Data on *T. cati* abundance were non-normally distributed and zero inflated. Generalized linear mixed models with negative binomial regression were used to analyze the relationship between *T. cati* abundance and age, sex, presence of cestodes, and lynx population density index. The year the lynx was hunted or had died and the GMD were included as random effects. Because *T. cati* abundance and age had a significant relationship with a second degree polynomial involving age squared, both age and age squared were fitted as explanatory variables into the analysis of variation of worm abundance.

The aggregation parameter *k* was calculated with the model $\left(k=\frac{\mu^{2}-\frac{\sigma^{2}}{N}}{\left(\sigma^{2}-\mu \right)}\right)$, which was originally described by Fisher (1941) and Bliss and Fisher (1953), and further modified by Elliott (1971) by including sample size (*N)*, which gives better estimation for the *k* (Buhat et al., 2020). The variance in the model is σ and the mean is μ. If *k* approaches zero and is less than one, it is an indicator of a highly aggregated parasite population (Shaw and Dobson, 1995; Buhat et al., 2020).

The statistical analyses were performed with SPSS 27 (IBM Corporation, New York, USA).

## 3 Results

### 3.1 *Toxocara cati* prevalence

**Fig. 1:**
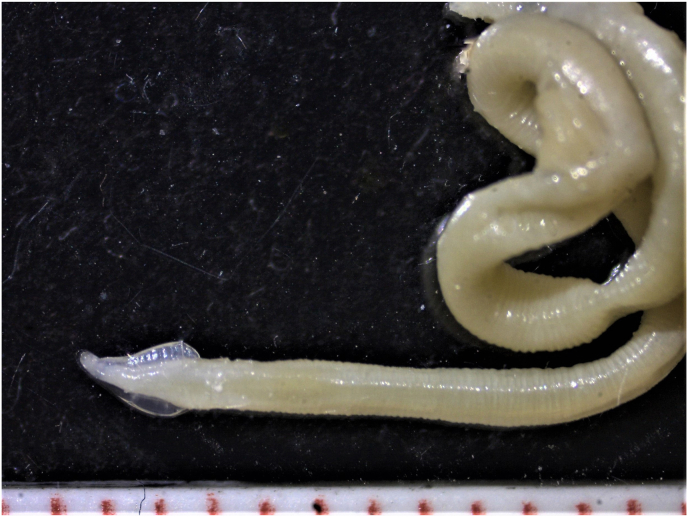
The head, the cephalic alae and the body of the *Toxocara cati* found from female Eurasian lynx (*Lynx lynx*) from Finland. The measurement scale has red marks at 1 mm intervals. (For interpretation of the references to colour in this figure legend, the reader is referred to the Web version of this article.)

Parasitic worms were found in 2565 (93.1%, 95% confidence interval (CI) 92.1–94.0) of the 2756 examined Eurasian lynx. *Toxocara cati* infection (Fig. 1) was detected in 2324 of the lynx, yielding an apparent infection prevalence of 84.3% (95% CI 82.9–86.0) (Table 1). Cestode infection was detected in 2023 (74.0%, 95% CI 72.4–75.7) of the lynx.

**Table 1:** Number of examined Eurasian lynx (*Lynx lynx*), number of *Toxocara cati* positive lynx, *T. cati* prevalence, abundance (mean, standard deviation), intensity (mean, standard deviation, median, range), and parameter *k* as an aggregation indicator.

	Number of lynx examined	Number of *Toxocara cati* positive lynx	Prevalence (%)	Abundance, mean	Abundance, standard deviation	Intensity, mean	Intensity, standard deviation	Intensity, median	Intensity, range	Parameter *k*
Age ∼6 months	645	543	84.2	18.4	28.0	22.0	29.2	11.0	1–218	0.444
Age 1–2 years	1276	1096	85.9	21.0	26.9	24.3	26.9	15.5	1–213	0.628
Age 3 years and over	835	685	82.0	12.9	19.7	15.7	20.7	8.0	1–183	0.442

Female	1138	959	84.3	18.0	25.6	21.2	25.7	13.0	1–213	0.511
Male	1618	1365	84.4	17.9	25.4	21.2	26.2	12.0	1–218	0.517

Total	2756	2324	84.3	18.0	25.5	21.2	26.0	12.0	1–218	0.513

**Fig. 2:**
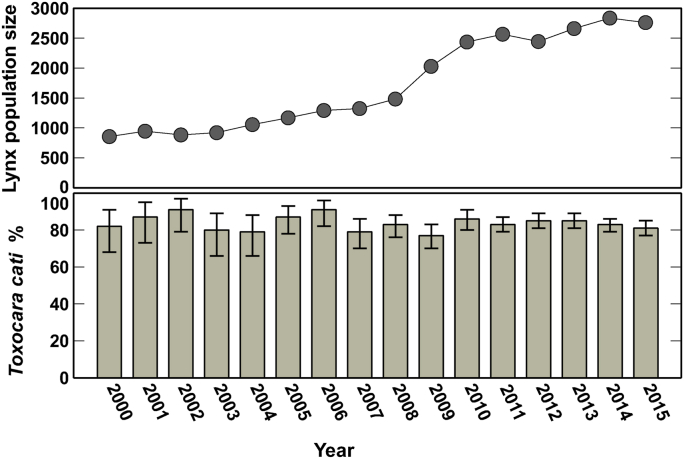
The estimated Eurasian lynx (*Lynx lynx)* population size and *Toxocara cati* prevalence (%, 95% confidence interval) in Finland, by year.

*Toxocara cati* prevalence was high throughout the years, varying from 77.2% (95% CI 70.4–84.0) in 2009 to 92.3% (95% CI 84.8–99.8) in 2006 (Fig. 2). The prevalence was over 80% in most of the years and did not vary statistically significantly between the years *(p* = 0.905), although the Eurasian lynx population size increased (Fig. 2).

The proportion of Eurasian lynx infected with *T. cati* was high in all age groups (Table 1). There was a significant negative association between presence of *T. cati* and age (*Coef*. = −0.070, *p* = 0.002, standard error (*SE*) = 0.023, *df* = 1, *F* = 93.73). The prevalence of *T. cati* did not differ statistically significantly between sexes (*p* = 0.778). There was a significant positive relationship between the presence of *T. cati* and the presence of cestodes (*Coef.* = 1.172, *p <* 0.001, *SE* = 0.121, *df* = 1, *F* = 93.73). Lynx density index did not appear to be a significant factor (*p* = 0.812). No interactions were detected between the investigated factors.

### 3.2 *Toxocara cati* abundance and intensity

The mean *T. cati* abundance was 18.0 (standard deviation = 25.5), and the mean *T. cati* intensity was 21.2 (standard deviation = 26.0) (Table 1). The parameter *k* for parasite aggregation was 0.513 indicating highly aggregated parasite distribution.

There was a significant negative association between age of the lynx and *T. cati* abundance (*Coef*. = −0.191, *p* < 0.001, *SE* = 0.028, *df* = 1, *F* = 45.80). The worm abundance was significantly lower amongst older lynx. Lynx density did not have a significant effect on worm abundance (*p* = 0.434). The variance of the random effect for the years was different from zero, indicating the years were different in regards to *T. cati* abundance.

**Fig. 3:**
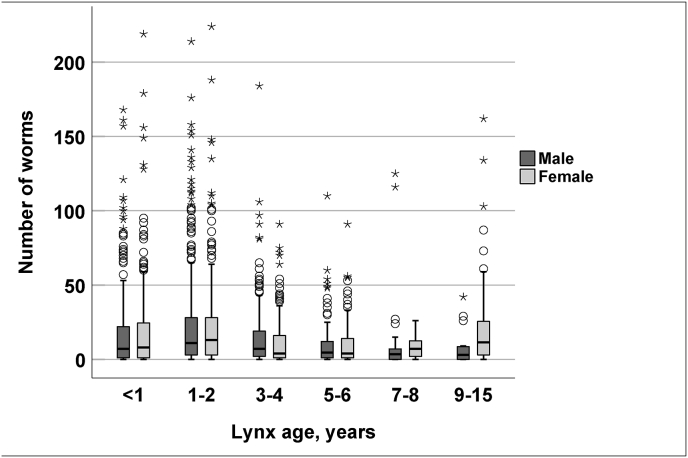
Number of *Toxocara cati* worms in Eurasian lynx (*Lynx lynx*) in Finland, by age and sex. Median, first and third quartiles, and minimum and maximum are shown.

Age squared and *T. cati* abundance were significantly positively associated (*Coef.* = 0.008, *p* = 0.011, *SE* = 0.003, *df* = 1, *F* = 17.71). There was an interaction with *T. cati* abundance, age squared, and sex (*p* = 0.009, *SE* = 0.003, *df* = 1, *F* = 11.13). Females aged 9–15 years had a significant positive relationship with *T. cati* abundance (*Coef.* = 1.298, *p* < 0.001, *SE* = 0.374, *df* = 5, *F* = 3.53), while the abundance was not related to age in males. Among the total of 67 lynx aged 9–15 years, the mean abundance was 26.0 (range 0–162) in the 40 females and 7.5 (range 0–42) in the 27 males (Fig. 3).

There was a positive relationship between *T. cati* abundance and presence of cestodes, indicating a higher probability of co-infection when *T. cati* abundance was higher (*Coef.* = 0.377, *p* < 0.001, *SE* = 0.067, *F* = 31.93).

## 4 Discussion

This longitudinal study using exceptionally large data gives extensive new insight to the host-parasite dynamics of wild, free-ranging Eurasian lynx and the zoonotic nematode *T. cati*. Unexpectedly, *T. cati* prevalence was not dependent on lynx population size, but instead remained high across the years – while the lynx population size tripled (Fig. 2). *Toxocara cati* abundance was not dependent on lynx population density either. These observations represent an important addition to our understanding on parasites in relation to host population size, as they contrast what is expected based on established literature (May and Anderson, 1979; Dobson and Hudson, 1992; Arneberg et al., 1998). While the observations are supported by the collected data, they may be partly explained by the lynx density not reaching a relevant threshold or by the territorial behavior of the lynx. With the exception of females with cubs, adult Eurasian lynx live a solitary life, have large home ranges, and display intrasexual territoriality whereby especially adult males have a tendency to avoid conspecifics (Schmidt et al., 1997; Linnell et al., 2001; Herfindal et al., 2005; Breitenmoser-Würsten et al., 2007).

*Toxocara cati* prevalence remained high throughout the study period and varied relatively little by year (Fig. 2). This might be explained either by *T. cati* being co-introduced with the lynx to an empty niche, or by a high infection pressure. Co-introduction of parasites and their hosts is a common phenomenon when hosts are invasive, introduced, or immigrating to new or empty niches (Williamson and Griffiths, 1996; Laurimaa et al., 2016; Lesniak et al., 2017). On the other hand, *T. cati* life cycle is well-established and maintained in Finland with contributions by domestic cats (Kauhala et al., 2015; Näreaho et al., 2012). While little is known on the level of local environmental contamination with *T. cati* eggs as well as on the prevalence of the infection in relevant paratenic hosts, the parasite is endemic and can infect the dispersing lynx.

*Toxocara cati* infection was highly prevalent in Eurasian lynx of all age groups and both sexes (Table 1). Our hypotheses of age-dependent and male-biased parasitism were not supported. Similar results have been reported earlier from Finland: in a cross-sectional study the proportion of individuals harboring *T. cati* worms was found to be almost the same in sub-adult and adult lynx, and in lynx of both sexes (Deksne et al., 2013).

The abundance of *T. cati* was significantly lower in older lynx and, interestingly, older females aged 9–15 years harbored a significantly higher number of *T. cati* nematodes than males of the same age group (Fig. 3). The mean intensity of *T. cati* was lower in lynx in the oldest age group than in younger lynx (Table 1). This is in contrast to the findings from Estonia, where no significant difference in *T. cati* intensity was observed between young and adult lynx (Valdmann et al., 2004). For interpreting this and other comparisons, it should be emphasized that our age group categories differ from other studies; we had sufficient data for biologically relevant age groups. Eurasian lynx in Finland typically start to reproduce at the age of three years (Holmala, unpublished). Our sample included a good number of older individuals, while one age group that was not included in our study was young cubs.

The parasites were strongly aggregated, which in line with previous studies that have reported parasite aggregation of intestinal worms in mammalian hosts (Newey et al., 2005; Saeed and Kapel, 2006). The differences between age groups and between sexes were relatively small. We were unable to analyze the effect of seasonality on aggregation because the sampling was largely linked to hunting seasons.

The detection of parasitic worms relied on their visibility, and smaller parasites may have been missed. Moreover, the carcasses were frozen, and the freezing and thawing may have affected the success of detecting all parasitic worms. Our results may thus be an underestimation of prevalence, abundance and intensity; however, as the procedures were the same throughout the study period and for all the animals investigated, the data are comparable across years and between individual hosts.

Interestingly, Eurasian lynx with higher *T. cati* abundance were more likely to be co-infected with cestodes. Similar results have been found in domestic cats in Finland: cats that shed cestode eggs in their feces had higher odds of shedding *Toxocara* or *Toxascaris* eggs than cats that did not shed cestode eggs in their feces (Näreaho et al., 2012). Several cestode species have been described in the Eurasian lynx, including *Taenia* spp. and *Mesocestoides* sp. (Deksne et al., 2013; Lavikainen et al., 2013; Haukisalmi et al., 2016).

*Toxocara cati* is a zoonotic parasite that can cause toxocariasis in humans (Despommier, 2003; Fisher, 2003; Holland, 2017). Compared with *T. canis*, *T. cati* has been receiving less attention both as a parasite of veterinary importance and as a zoonotic parasite (Fisher, 2003; ESCCAP, 2021; Maciag et al., 2022). It is worth emphasizing that routine serological methods cannot distinguish between infections caused by *T. canis* and *T. cati*, and the public health importance of *T. cati* is not fully elucidated (Fisher, 2003; Poulsen et al., 2015; Maciag et al., 2022).

In felids, most *T. cati* infections are subclinical. In domestic cats, heavy *T. cati* infection can cause clinical signs including pot-bellied appearance, diarrhea, vomiting and inappetence (ESCCAP, 2021; Ursache et al., 2021). *Toxocara* spp. has been reported as the cause of death of two juvenile Eurasian lynx in Switzerland (Schmidt-Posthaus et al., 2002). It cannot be ruled out that the infections in Eurasian lynx could have potential effect on mortality and could have introduced bias in this study, but as most of the detected infections were light, they were unlikely to have predisposed the individuals to hunting or traffic accidents.

Our results indicate that Eurasian lynx contribute to the circulation of *T. cati* in Finland, which has both potential public health and animal health importance. This highlights that free-ranging wildlife species should not be forgotten in the One Health investigations and approaches targeting zoonotic pathogens such as *T. cati*.

## 5 Conclusion

The results of this study showed that *T. cati* has been highly prevalent and abundant amongst Eurasian lynx in Finland over 16 years, while the host population size increased substantially. Our work yielded new information about the host-parasite dynamics and about the contribution of free-ranging Eurasian lynx to the circulation of the zoonotic parasite.

## Funding

This study was supported by 10.13039/501100004022Jenny and Antti Wihuri Foundation (00200414, 09.10.2020), The 10.13039/501100003125Finnish Cultural Foundation (00191157, 27.02.2019), and Societas pro Fauna et Flora Fennica (07.12.2018). The funders had no role in study design, in the collection, analysis and interpretation of data, in the writing of the report and in the decision to submit the article for publication.

## Data availability

The parasites and the raw data were gathered and provided for this study by the Natural Resources Institute Finland. The data were used under the license with restriction, and are not available for the public. All relevant data are provided in this article.

## Declarations of competing interest

The authors declare that there is no conflict of interest.
